# An Evaluation of Firearm Injuries in the Emergency Department

**DOI:** 10.7759/cureus.20555

**Published:** 2021-12-20

**Authors:** Meltem Songür Kodik, Öykü Bakalım Akdöner, Zeyyat Cüneyt Özek

**Affiliations:** 1 Emergency Department, Ege University Faculty of Medicine, İzmir, TUR; 2 Emergency Department, Malatya Training and Research Hospital, Malatya, TUR; 3 Plastic and Reconstructive Surgery, Ege University Faculty of Medicine, İzmir, TUR

**Keywords:** forensic medicine, emergency medicine, trauma, gunshot wound, firearm injury

## Abstract

Introduction

Firearm injuries are a significant cause of mortality and morbidity. Our study aims to evaluate the injury patterns, results of imaging studies, treatment methods, outcomes, and mortality rates of patients who were admitted to the emergency department with firearm injuries.

Methods

Our study was designed as a retrospective descriptive study. To this end, adult patients who were admitted to our hospital with gunshot wounds between January 1, 2017, and July 31, 2021, were screened. The files of 527 patients who were admitted with gunshot wounds were analyzed. A total of 30 patients were excluded from the study due to missing data. Statistical analyses were performed using the data of a total of 497 patients. Independent variables of the study included sex, age, systolic blood pressure (SBD), diastolic blood pressure (DBD), pulse, respiratory rate, Glasgow Coma Scale (GCS) score, range of shot, injury site, X-ray, cranial CT, thorax CT, abdominal CT, and extremity CT angiography findings, and the need for treatment and referral. Mortality was the dependent variable of the study. A logistic regression model was created to predict factors affecting the survival of the patients who were admitted to the emergency department with gunshot wounds and to identify the independent variables affecting survival. A p-value of <0.05 was considered sufficient for significance.

Results

The majority of patients who were admitted to the emergency department due to gunshot wounds were male and the median age of the patients was 32 years (18-70 years). The comparison of the descriptive characteristics with respect to survival revealed that the systolic and diastolic blood pressures and GCS scores of the deceased patients were significantly lower than those of the survivors. The rate of shooting at short range was significantly higher in the deceased patients when compared to that of the survivors. In addition, the rate of the need for surgical intervention and the incidence of pneumocephaly in cranial CT were higher in the deceased patients than in the survivors. Significantly higher rates of deceased patients required referral to neurosurgery and thoracic surgery clinics than survivors. The patients who were referred to the thoracic surgery clinic had an increased death rate by 29-fold and the patients who were referred to the thoracic surgery clinic had an increased death rate by about nine-fold. On the other hand, the probability of death was reduced by about half when the GCS scores of the patients were higher.

Discussion

We evaluated GCS in our patient group and determined a significantly lower score in the patients who did not survive, which agrees with the findings of other studies. Patients with higher SBD and DBD showed a higher probability of survival, which agrees with the results in other studies. Most patients were shot from their extremities and none had died while the death rate was significantly higher in the patients who suffered injuries to the head or neck. The patients with pneumocephalus had a very low chance of survival. Compared to wound care and dressing, patients who received surgical treatment were more likely to die as these patients had more critical injuries.

Conclusion

Although most injuries were to the extremities, there were no mortalities in the cohort of patients referred to orthopedics. The patients who suffered injuries to the head/neck had the highest mortality rate.

## Introduction

Firearm injuries are a significant cause of mortality and morbidity. According to the data from the World Health Organization, approximately 251,000 (95% CI: 1950000-276,000) gunshot wound deaths occurred worldwide in 2016. Gunshot wounds have attracted attention as a public health problem globally, with an age-standardized mortality rate of 3.4 (95% CI: 2.6-3.7) per 100,000 people [1].

Deaths due to firearm injuries are common in the military field, but it has become common in the civilian population with the increasing number of firearms owned by civilians. Thus, the number of patients admitted to the emergency services of civilian hospitals with gunshot wounds is increasing. Deaths due to gunshot wounds in the United States were reported to be 39,707 in 2019, leading to a rate of 12.1 per 100,000 [2].

Although death rates after gunshot wounds are higher than other types of injuries such as motor vehicle accidents, a significant number of patients are treated and discharged from hospitals after suffering gunshot wounds.

Our study aims to evaluate the injury patterns, results of imaging studies, treatment methods, and outcomes and mortality rates of the patients who were admitted to the emergency department with firearm injuries.

## Materials and methods

Our study was designed as a retrospective descriptive study following the Strengthening the Reporting of Observational Studies in Epidemiology (STROBE) guidelines. To this end, adult patients with gunshot wounds who were admitted between January 1, 2017, and July 31, 2021, to our hospital as the primary care center were screened.

The files of 527 patients who were admitted with gunshot wounds were analyzed. A total of 30 patients were excluded from the study due to missing data. Statistical analyses were performed using the data of a total of 497 patients.

The independent variables of the study included sex, age, systolic blood pressure (SBD), diastolic blood pressure (DBD), pulse, respiratory rate, Glasgow Coma Scale (GCS) score, range of shot, injury site, X-ray, cranial CT, thorax CT, abdominal CT, and extremity CT angiography findings, and the need for treatment and referral. The dependent variable of the study was mortality.

The first vital parameters and GCS of the patients were measured and recorded upon their admission to the emergency department and fluctuations in these parameters were not recorded. The type of weapon that caused the injury was recorded in terms of long- and short-barreled. The injury sites were identified as head and neck, thorax, abdomen, upper extremity, lower extremity, and multiple sites.

Foreign body and fracture were specified using the X-ray findings. Pneumocephalus, intracranial hemorrhage, and foreign body and skull fracture were specified using the cranial CT findings. Pneumothorax, hemothorax, laceration in the lung, and foreign body in the thorax were specified using the thorax CT findings. Liver laceration, spleen laceration, intestinal perforation, free fluid and air in the abdomen, foreign body in the abdomen, and kidney laceration were specified in using the abdominal CT findings. Vascular injury, foreign body in extremity, and extremity bone fractures were specified using the extremity CT angiography findings. Treatment was analyzed in terms of two categories comprising surgery and wound care. Other data were obtained and recorded using the patient files.

The data of patients who underwent neurosurgery, otolaryngology, ophthalmology, plastic surgery, orthopedics, cardiovascular surgery, urologic surgery, thoracic surgery, or general surgery consultations were recorded.

Approximately 60 adult patients are annually admitted to the emergency department with gunshot wounds. We planned to evaluate 300 patients with patient file scanning of five years. However, the patient file scan led to exceeding the planned number of patients.

Statistical analysis

Statistical analyses were performed using the Statistical Package for the Social Sciences (SPSS) program, version 26.0 (IBM Corp, Armonk, NY). The results were presented in terms of median, minimum, and maximum values for numerical variables and frequency and percentage values for categorical data. All statistical results were presented using tables.

The fitness of the variables to normal distribution was evaluated using the Kolmogorov-Smirnov test. The chi-square test and Fisher's exact test were used to compare the categorical variables. Additionally, the Mann-Whitney U test was used to compare the non-parametric variables. A logistic regression model was created to predict the factors affecting the survival of the patients who were admitted to the emergency department with gunshot wounds and to identify the independent variables affecting survival. A p-value of <0.05 was considered sufficient for statistical significance.

## Results

The majority of the patients who were admitted to the emergency department due to gunshot wounds were male and the median age of the patients was 32 years (18-70 years) (Table 1).

**Table 1:** Descriptive characteristics of the injured patients. SBD: systolic blood pressure; DBD: diastolic blood pressure.

Descriptive characteristics of the injured patients			
Sex	Female (n, %)	52	10.5
	Male (n, %)	445	89.5
Age (median, min-max)		32	18-70
SBD (median, min-max)		122	0-190
DBD (median- min-max)		78	0-119
Pulse (median- min-max)		90	0-160
Respiratory rate (median- min-max)		16	0-25
Glasgow Coma Scale score (median- min-max)		15	3-16
Outcome (n, %)	Discharged alive	474	95.4
	Dead	23	4.6

The comparison of the descriptive characteristics of the patients in terms of survival revealed that the systolic and diastolic blood pressures and GCS scores of the deceased patients were significantly lower than those of the survivors (Table 2).

**Table 2:** Comparison of the descriptive characteristics in terms of survival. F: Fisher’s exact test; Z: Mann-Whitney U test; SBD: systolic blood pressure; DBD: diastolic blood pressure.

		Outcome					
		Discharged alive		Dead		Test value	p-value
Sex	Female (n, %)	49	10.3	3	13.0	0.171^F^	0.723
	Male (n, %)	425	89.7	20	87.0		
Age (median, min-max)		32	18-70	31	18-60	0.703^Z^	0.482
SBD (median, min-max)		124	69-190	86	0-153	4.985^Z^	<0.001
DBD (median, min-max)		78	30-119	54	0-82	5.747^Z^	<0.001
Pulse (median, min-max)		90	11-145	100	0-160	1.187^Z^	0.235
Respiratory rate (median, min-max)		16	12-25	14	0-22	1.567^Z^	0.117
Glasgow Coma Scale score (median, min-max)		15	5-15	3	3-15	16.032^Z^	<0.001

The rate of shooting at short range was significantly higher in the deceased patients when compared to that of the survivors. In addition, the rate of the need for surgical intervention and the incidence of pneumocephaly in cranial CT were higher in the deceased patients than in the survivors (Table 3).

**Table 3:** Comparison of the trauma characteristics in terms of survival. Each subscript letter denotes a new subset of outcome categories with column proportions that do not differ significantly from each other at the 0.05 level. da: discharged alive; d: dead; C: chi-square test; F: Fisher’s exact test; CT: computed tomography.

		Outcome							
		Discharged alive		Dead					
		n	% (da)	n	% (d)	Test value			p-value
Range of shot	Short	265	55.9	19	82.6	6.386^C^			0.012
	Long	209	44.1	4	17.4				
Injury site	Head and neck	32_a_	6.8	12_b_	52.2	56.169^F^			<0.001
	Thorax	32_a_	6.8	4_a_	17.4				
	Abdomen	21_a_	4.4	2_a_	8.7				
	Lower extremity	271_a_	57.2	0_b_	0.0				
	Upper extremity	51_a_	10.8	0_a_	0.0				
	Multi-site injury	67_a_	14.1	5a	21.7				
X-ray	Foreign body	82	36.3	1	100.0	1.853^F^			0.546
	Fracture	103	45.6	0	0.0				
	Foreign body and fracture	41	18.1	0	0.0				
Cranial CT	Pneumocephalus	2_a_	4.5	8_b_	61.5	25.151^F^			<0.001
	Intracranial hemorrhage	5_a_	11.4	2_a_	15.4				
	Foreign body in the head	26_a_	59.1	0_b_	0.0				
	Skull fracture	11_a_	25.0	3_a_	23.1				
Thorax CT	Pneumothorax	7	13.0	1	25.0	3.031^F^			0.583
	Hemothorax	10	18.5	0	0.0				
	Laceration in the lung	11	20.4	1	25.0				
	Foreign body in the thorax	13	24.1	0	0.0				
	Pneumothorax + hemothorax + rib fracture + laceration	13	24.1	2	50.0				
Abdominal CT	Liver laceration	9	18.0	1	25.0	3.948^F^			0.322
	Spleen laceration	4	8.0	0	0.0				
	Intestinal perforation	6	12.0	1	25.0				
	Free fluid and air in the abdomen	12	24.0	2	50.0				
	Foreign body in the abdomen	19	38.0	0	0.0				
	Kidney laceration	0	0.0	0	0.0				
Extremity CT angiography	Vascular injury	43	42.6	1	100.0	1.868^F^			0.539
	Foreign body in extremity	47	46.5	0	0.0				
	Extremity bone fracture	11	10.9	0	0.0				
Treatment	Surgical	192	40.5	16	69.6	7.611^C^			0.006
	Wound care and dressing	282	59.5	7	30.4				

Significantly higher rates of deceased patients required referral to neurosurgery and thoracic surgery clinics than the survivors (Table 4).

**Table 4:** Comparison of the need for referral in terms of survival. da: discharged alive; d: dead; C: chi-square test; F: Fisher’s exact test.

		Outcome new					
		Discharged alive		Dead			
		n	% (da)	n	% (d)		
Referred to neurosurgery	Absent	430	90.7	9	39.1	56.630^F^	<0.001
	Present	44	9.3	14	60.9		
Referred to otolaryngology	Absent	451	95.3	20	87.0	3.228^F^	0.103
	Present	22	4.7	3	13.0		
Referred to ophthalmology	Absent	458	96.8	22	95.7	0.097^F^	0.538
	Present	15	3.2	1	4.3		
Referred to plastic surgery	Absent	372	78.5	19	82.6	0.223^F^	0.797
	Present	102	21.5	4	17.4		
Referred to orthopedic surgery	Absent	164	34.6	23	100.0	39.978^C^	<0.001
	Present	310	65.4	0	0.0		
Referred to cardiovascular surgery	Absent	355	74.9	17	73.9	0.011^C^	0.916
	Present	119	25.1	6	26.1		
Referred to general surgery	Absent	421	88.8	19	82.6	0.833^F^	0.321
	Present	53	11.2	4	17.4		
Referred to urologic surgery	Absent	457	96.4	23	100.0	0.854^F^	0.355
	Present	17	3.6	0	0.0		
Referred to thoracic surgery	Absent	424	89.5	15	65.2	12.498^F^	0.003
	Present	50	10.5	8	34.8		

A logistic regression model was created using the GCS score and the need for referral to thoracic surgery and neurosurgery with reference to the variables that were significant in pairwise comparisons. The model's Nagelkerke R square was 0.749, sensitivity was 99.6%, and specificity was 73.9. The patients who were referred to the thoracic surgery clinic had an increased death rate by 29-fold and the patients who were referred to the thoracic surgery clinic had an increased death rate by about nine-fold. On the other hand, the probability of death was reduced by about half when the GCS scores of the patients were higher (Table 5).

**Table 5:** Logistic regression analysis output showing the factors affecting survival.

	B	Wald	Sig.	Exp(B)	95% CI for EXP(B)	
					Lower	Upper
Glasgow Coma Scale score	−0.634	35.498	<0.001	0.531	0.431	0.654
Referred to thoracic surgery (present vs. absent)	3.378	10.240	0.001	29.309	3.702	232.023
Referred to neurosurgery (present vs. absent)	2.158	7.793	0.005	8.654	1.902	39.382
Constant	5.574	20.512	<0.001	263.402		

## Discussion

Gunshot wounds are one of the most complex injuries among penetrating injuries. Having high kinetic energy, they have higher mortality and morbidity rates than other blunt and sharps object injuries. This is mainly attributable to the high amount of energy transferred to the tissue and larger affected area [3]. The damage is proportional both to the energy transfer and to the biological characteristics and energy distribution of the tissue. This study evaluates the effect of injury patterns, results of imaging studies, treatment methods, and complications on the mortality rates of the patients who were admitted to our emergency department with gunshot wounds. We determined a mortality rate of 4.6% in the study group and 87% of the group was composed of males. Males suffer from firearm wounds more frequently (above 80%), leading to greater numbers of deceased males [4-7].

Several scoring systems are used to assess the prognosis and mortality in patients with firearm wounds such as Triage Revised Trauma Score (T-RTS), GCS, and Injury Severity Score (ISS). We evaluated GCS in our patient group on admission and determined a significantly lower score in the patients who did not survive (median GCS = 3), which is in line with the findings of other studies, which revealed GCS values that were typically lower than 5 for the non-surviving groups [7,8]. Patients with higher SBD and DBD showed a higher probability of survival in our study, which agrees with the results found by Saylam et al. [3].

Gunshot wounds were predominantly located at extremities and the mortality was high in the case of injuries to the thorax, head, or neck [7,9,10]. In this study, most patients were shot from their extremities and none had died, while the death rate was significantly higher in the patients who suffered injuries to the head or neck (p < 0.001). Studies have also linked gunshot injuries to the abdomen to high mortality. One such study was carried out by Saylam et al., who linked abdominal bleeding and injuries to the small intestine, colon, and stomach to higher mortality rates [3]. Moreover, the researchers associated subarachnoid hemorrhage, cerebral parenchymal contusion, and pneumocephalus with a lower chance of survival in the cases of head/neck injuries [3]. We discovered that patients with pneumocephalus had a very low chance of survival (p < 0.001).

Surgical treatment is very common for penetrating traumas and is characterized by the location of the injury. Compared to wound care and dressing, patients who received surgical treatment were more likely to die as these patients have more critical injuries. Liebenberg et al. determined that among the patients receiving surgical treatment, the mortality rate was 86.4% for the patients with a GCS score of 3-8, while Martins et al. found a rate of 48.9% for a similar GCS score range [11,12].

Most firearm injuries were to the extremities. Thus, most referrals were to orthopedic surgery, followed by the referrals to cardiovascular surgery [7], which agrees with our findings. Although the patients who were referred to the orthopedics had a higher chance of survival (p < 0.001), the rate of mortality was considerably high in the patients who underwent neurosurgical or thoracic surgery. The application of logistic regression to identify the role of independent variables in survival status revealed that thoracic and neurosurgical treatment had increased the risk of mortality by 29-fold and 9-fold, respectively, while higher GCS scores reduced the risk of mortality by half. Turgut et al. found that the number of deceased patients who were referred to the neurosurgery department was critically higher than other referrals [9]. Moreover, penetrating thoracic injuries were likely to cause destructive trauma to multiple organ systems, thus increasing lethality. Therefore, these patients should be evaluated emergently for severe injury even when they have hemodynamic stability on presentation and intervened with immediate surgical management to increase their chance of survival [13].

Limitations

The main limitation of this study is its single-centeredness and retrospective nature, thus limiting the generalizability of the results. In addition, the study did not consider the type of weapon and bullet velocity, which are important factors when determining the extent of the damage to the tissue/organ. Moreover, considering the critical importance of the first hours after gunshot injuries, recording the time elapsed from hospitalization to death could have improved our analysis.

## Conclusions

Gunshot injuries are a critical public health issue and cause disability or premature death in numerous patients. A scientific approach is needed to determine the predictive factors and develop effective treatment methods to reduce the rate of mortality. We found that most wounds were to the extremities; however, the patients who suffered injuries to the head/neck had the highest mortality rate.
